# Fish Red Blood Cells Modulate Immune Genes in Response to Bacterial Inclusion Bodies Made of TNFα and a G-VHSV Fragment

**DOI:** 10.3389/fimmu.2019.01055

**Published:** 2019-05-22

**Authors:** Sara Puente-Marin, Rosemary Thwaite, Luis Mercado, Julio Coll, Nerea Roher, Maria Del Mar Ortega-Villaizan

**Affiliations:** ^1^Departamento de Bioquímica y Biología Molecular, Instituto de Biología Molecular y Celular (IBMC), Universidad Miguel Hernández (UMH), Elche, Spain; ^2^Departamento de Bioquímica y Biología Molecular, Instituto de Investigación, Desarrollo e Innovación en Biotecnologîa Sanitaria de Elche (IDiBE), Universidad Miguel Hernández (UMH), Elche, Spain; ^3^Department Biologia Cellular, Fisiologia Animal i Immunologia, Institut de Biotecnologia i de Biomedicina (IBB), Universitat Autònoma de Barcelona (UAB), Barcelona, Spain; ^4^Grupo de Marcadores Inmunológicos, Laboratorio de Genética e Inmunología Molecular, Instituto de Biología, Pontificia Universidad Católica de Valparaíso (PUCV), Valparaíso, Chile; ^5^Departamento de Biotecnología, Instituto Nacional de Investigación y Tecnología Agraria y Alimentaria (INIA), Madrid, Spain

**Keywords:** erythrocytes, red blood cells, bacterial inclusion bodies, TNFα, VHSV glycoprotein G, immune response

## Abstract

Fish Red-Blood Cells (RBCs) are nucleated cells that can modulate the expression of different sets of genes in response to stimuli, playing an active role in the homeostasis of the fish immune system. Nowadays, vaccination is one of the main ways to control and prevent viral diseases in aquaculture and the development of novel vaccination approaches is a focal point in fish vaccinology. One of the strategies that has recently emerged is the use of nanostructured recombinant proteins. Nanostructured cytokines have already been shown to immunostimulate and protect fish against bacterial infections. To explore the role of RBCs in the immune response to two nanostructured recombinant proteins, TNF**α** and a G-VHSV protein fragment, we performed different *in vitro* and *in vivo* studies. We show for the first time that rainbow trout RBCs are able to endocytose nanostructured TNF**α** and G-VHSV protein fragment *in vitro*, despite not being phagocytic cells, and in response to nanostructured TNF**α** and G-VHSV fragment, the expression of different immune genes could be modulated.

## Introduction

Fish red blood cells (RBCs) are nucleated cells that contain organelles in their cytoplasm unlike those of mammals (1). Apart from their well-known role in gas exchange, recently a set of new biological roles for nucleated RBCs related to the immune response have been reported. Nucleated RBCs are able to phagocytose and act as antigen presenting cells (2, 3). They can respond to different pathogen associated molecular patterns (PAMPs), modulate leukocyte activity, release cytokine-like factors (4, 5) and lately they have been implicated in the response to viral infections [reviewed in Nombela and Ortega-Villaizan (6)].Considering all of these findings, the potential role of RBCs in the immune system of fish takes on a new, interesting perspective.

To date, one of the best strategies for preventing and controlling viral diseases in aquaculture is DNA vaccination. However, it remains unclear which mechanisms are responsible for this protection (7). The search for new, safe and effective vaccines has become a priority in this field. Among fish viral diseases, viral hemorrhagic septicaemia (VHS) is a lethal infectious fish disease caused by viral hemorrhagic septicaemia virus (VHSV), which affects over 50 species of fish, freshwater and marine, in the northern hemisphere (8).

As an alternative to overcome the safety problems associated to live attenuated or DNA vaccines, bacterial inclusion bodies (IBs) nanostructured recombinant proteins have been presented as a new option for vaccination (9). IBs are *per se* strong stimulants of the fish immune system and have a set of characteristics which make them an attractive alternative: they are mechanically stable, production is scalable and cost-effective, they are non-toxic biomaterials and are composed of recombinant proteins. The latter means they are an adaptable prototype, which could be a good platform for vaccination against a wide range of diseases (9, 10). Such nanostructured recombinant proteins have already been shown to protect fish against bacterial infection (9).

In this paper, we show for the first time the response of rainbow trout RBCs *in vitro* and *in vivo* to two different nanostructured recombinant proteins, recombinant rainbow trout tumor necrosis factor alpha protein (IB^TNFα^) and recombinant fragment 16 of the glycoprotein G of VHSV (11) (IB^frg16G−VHSV^). In response to recombinant protein IBs, RBCs were able to modulate the expression of interferon related genes, the myxovirus resistance (*mx*) gene and genes related to antigen presentation (cluster of differentiation 83 [*cd83*], major histocompatibility class I [*mhcI*] and major histocompatibility class II [*mhcII*]). Genes related to antioxidant response (natural killer enhancing factor [*nkef*] and glutathione S-transferase pi 1 gene [*gstp1*] and cytokines (interleukin 1β [*il1*β], interleukin 12β [*il12*β], interleukin 6 [*il6*], interleukin 2 [*il2*], and interleukin 8 [*il8*]) were also modulated. Interestingly, IB^TNFα^ mostly down-regulated *in vitro* and *in vivo* immune genes expression in RBCs meanwhile IB^frg16G−VHSV^ mainly showed an up- regulation trend.

## Materials and Methods

### Production of IBs, Purification, Quantification, and Fluorescent Labeling

Nanostructured proteins were produced in *E. coli* following the method described in Torrealba et al. (9) and Thwaite et al. (12). In short, *E. coli* transformed with the plasmid of interest was cultured in LB with the appropriate antibiotic and recombinant protein expression was induced at OD_550nm_ 0.5–0.8 with 1 mM IPTG (Panreac, Barcelona, Spain). IBs were isolated after 3 h additional incubation at 37°C via enzymatic and mechanical disruption of the cells according to Torrealba et al. (10), followed by sterility monitoring (12). Purified nanoparticles, named here IB^frg16G−VHSV^, IB^TNFα^ and IB^iRFP^ [an inclusion body made of a non-immunogenic phytochrome-based near infra-red fluorescent protein (iRFP) with the excitation/emission maxima at 690/713 nm (13)], were stored at −80°C until use. Quantification was performed by western blot using an anti-His-tag antibody (Genscript, Piscataway, NJ, USA) and calculating the protein concentration from a standard curve using Quantity One software (Biorad, Hercules, CA, the USA). For flow cytometry or confocal microscopy, IB^frg16G−VHSV^ and IB^TNFα^ were conjugated with fluorescent Atto-488 NHS ester (Sigma-Aldrich) following manufacturer's instructions.

### Animals

Juvenile rainbow trout (*Oncorhynchus mykiss*) were obtained from a commercial farm (Piszolla S.L., Cimballa Fish Farm, Zaragoza, Spain), and maintained at the University Miguel Hernandez (UMH) facilities at 14°C, fed daily with a commercial diet (Skretting, Burgos, Spain). Prior to experiments, fish ware acclimatized to laboratory conditions for 2 weeks. Separately, adult rainbow trout were maintained at the Universitat Autònoma de Barcelona (UAB) at 17 ± 1°C, fed daily with a commercial diet. The number of individuals used in each experiment is indicated by an “n” in each figure legend.

### Cell Cultures

Rainbow trout RBCs were obtained from peripheral blood of fish sacrificed by overexposure to tricaine (tricaine methanesulfonate, Sigma-Aldrich) (0.3 g/L). Peripheral blood was sampled from the caudal vein using insulin syringes (Nipro, Bridgewater, NJ, USA) as previously described (14). RBCs were purified by two consecutive density gradient centrifugations (7,206 g, Ficoll 1.007; Sigma-Aldrich). Purity of RBCs of 99.9% was estimated by optical microscopy (Figure S1). Purified RBCs were cultured with RPMI-1640 medium (Dutch modification) (Gibco, Thermo Fischer Scientific Inc., Carlsbad, CA) supplemented with 10% fetal bovine serum (FBS) gamma irradiated (Cultek, Madrid, Spain), 1 mM pyruvate (Gibco), 2 mM L-glutamine (Gibco), 50 μg/mL gentamicin (Gibco) and 2 μg/mL fungizone (Gibco), 100 U/mL penicillin and 100 μg/mL streptomycin (Sigma-Aldrich) at a density of 10^6^ cells/mL at 14°C.

### Uptake of IB^TNFα^ and IB^frg16G-VHSV^ by RBCs

RBCs cultures were treated with fluorescent IB^TNFα^ or IB^frg16G−VHSV^ at different concentrations and uptake was analyzed by flow cytometry using a FACSCanto™ cytometer (BD Biosciences, Madrid, Spain) (10.000 total events), at different times post-treatment. For dose-response evaluation, IBs at concentrations of 10, 20 and 50 μg/mL were added to RBCs cultures for 24 h. For time-course experiments, RBCs were treated with 80 μg/mL IB^TNFα^ or 160 μg/mL IB^frg16G−VHSV^ for 6, 24 and 48 h. After incubation with IBs, the medium was removed and RBCs were washed with phosphate-buffered saline (PBS). RBCs were then resuspended in 200 μL of RPMI 2% FBS for flow cytometry analysis.

In addition, confocal microscopy was performed to evaluate the uptake of IBs by RBCs. RBCs were incubated with 80 μg /mL of IB^TNFα^ or 160 μg /mL of IB^frg16G−VHSV^ for 24 h. Then, medium was removed and RBCs were washed as indicated above. The RBC nucleus was labeled with 10 μg/mL Hoechst (Sigma-Aldrich) and RBC membrane was stained with 5 μg/mL of CellMask (Thermo Fischer Scientific). Images were taken with a Zeiss LSM 700 microscope (Zeiss, Oberkochen, Germany) and analyzed with Imaris Software v8.2.1 (Bitplane, Zurich, Switzerland).

### RBCs Immune Response After *in vitro* Treatment With IB^TNFα^ or IB^frg16G-VHSV^

RBCs were treated *in vitro* with 50 μg/mL of each IB for 24 h. IB^iRFP^ was used as a control. After treatment, RBCs were resuspended in TRK lysis buffer (Omega Bio-Tek Inc., Norcross, GA, USA) and stored at −80°C until RNA extraction.

### RBCs Immune Response After *in vivo* Treatment With IB^TNFα^ or IB^frg16G-vhsv^

Juvenile rainbow trout (15–20 g) were treated by intravenous injection in caudal vein with 50 μL of IBs (5.5 mg/kg) or 50 μL of PBS. At 24 and 48 h post-injection fish were sacrificed by overexposure to tricaine. Peripheral blood was sampled as described above and resuspended in RPMI 10% FBS. Then, RBCs were Ficoll-purified as explained above. Purified RBCs were either resuspended in TRK lysis buffer and stored at −80°C until RNA extraction or fixed for immunofluorescence and flow cytometry, as described below.

In order to track the presence of IBs *in vivo*, IB^TNFα^ was monitored in peripheral blood and head kidney from IB^TNFα^ intravenously injected in caudal vein of rainbow trout by means of fluorescent microscopy using IN Cell Analyzer 6,000 Cell Imaging system (GE Healthcare, Little Chalfont, UK). Blood was extracted 3 h post-injection as described above. Head kidney was aseptically removed, placed in 24 well plates with RPMI 10% FBS and disaggregated with a Pasteur pipette and passed through a Falcon 40 μm nylon cell strainer (BD Biosciencies) using a plunger of a 5 ml syringe.

### RNA Isolation, cDNA Synthesis, RT-qPCR, and Gene Expression Analysis

RBCs total RNA was extracted as previously described (14) using E.Z.N.A.® Total RNA Kit (Omega Bio-Tek Inc.). DNAse treatment was performed in order to eliminate residual genomic DNA using TURBO™ DNase (Ambion, Thermo Fischer Scientific Inc.). Then cDNA synthesis and RT-qPCR was performed as described in Nombela et al (14). Primers and probes used are listed in Table 1. Gene expression was analyzed by means of the 2 ^−ΔCt^ or 2^−ΔΔCt^ (23) using 18S rRNA (Applied Biosystems, Thermo Fischer Scientific Inc.) as endogenous gene. Principal component analysis (PCA) and clustering heatmap of immune-gene expression data (2 ^−ΔCt^ or 2^−ΔΔCt^) were performed using Clustvis software (24). For PCA, unit variance scaling was applied to rows and singular value decomposition (SVD) with imputation was used to calculate principal components. For clustering heatmap, columns were collapsed by taking mean inside each group, rows were centered, and unit variance scaling was applied to rows; then, imputation was used for missing value estimation; and, both rows and columns were clustered using correlation distance and average linkage.

**Table 1:** List of primers and probes used.

**Gene**	**Forward primer**	**Reverse primer**	**Probe**	**Reference or accession number**
*tlr3*	ACTCGGTGGTGCTGGTCTTC	GAGGAGGCAATTTGGACGAA	CAAGTTGTCCCGCTGTCTGCTCCTG	(14)
*tlr9*	CCTGCGACACTTCCTGGTTT	GCCAGTGGTAAGAAGGAGGATCT	CAGACTTCCTGCGTGCCGGCC	(15, 16)
*ifn1*	ACCAGATGGGAGGAGATATCACA	GTCCTCAAACTCAGCATCATCTATGT	AATGCCCCAGTCCTTTTCCCAAATC	(14)
*mx1-3*	TGAAGCCCAGGATGAAATGG	TGGCAGGTCGATGAGTGTGA	ACCTCATCAGCCTAGAGATTGGCTCCCC	(16)
*il15*	TACTATCCACACCAGCGTCTGAAC	TTTCAGCAGCACCAGCAATG	TTCATAATATTGAGCTGCCTGAGTGCCACC	(14)
*nkef*	CGCTGGACTTCACCTTTGTGT	ACCTCACAACCGATCTTCCTAAAC		(14)
*gstp1*	CCCCTCCCTGAAGAGTTTTGT	GCAGTTTCTTGTAGGCGTCAGA		(14)
*hepcidin*	TCCCGGAGCATTTCAGGTT	GCCCTTGTTGTGACAGCAGTT		(14)
*trx*	AGACTTCACAGCCTCCTGGT	ACGTCCACCTTGAGGAAAAC		(14)
*il6*	ACTCCCCTCTGTCACACACC	GGCAGACAGGTCCTCCACTA	CCACTGTGCTGATAGGGCTGG	(17)
*il12β*	TGACAGCCAGGAATCTTGCA	GAAAGCGAATGTGTCAGTTCAAA	ACCCAACGACCAGCCTCCAAGATG	(17)
*tnfα*	AGCATGGAAGACCGTCAACGAT	ACCCTCTAAATGGATGGCTGCTT	AAAAGATACCCACCATACATTGAAGCAGATTGCC	(18)
*il8*	AGAGACACTGAGATCATTGCCAC	CCCTCTTCATTTGTTGTTGGC	TCCTGGCCCTCCTGACCATTACTGAG	(17, 19)
*il1β*	GCCCCCAACCGCCTTA	CAGTGTTTGCGGCCATCTTA	ACCTTCACCATCCAGCGCCACAA	(17)
*il2*	GTTGCAGCATTGGCCTGTT	TGTTCTCCTTATCAATCGTCTTTTGT	CAACACCACATCAGCATGACTGCCAC	NM_001164065.2
*cd83*	TTGGCTGATGATTCTTTCGATATC	TGCTGCCAGGAGACACTTGT	TCCTGCCCAATGTAACGGCTGTTGA	(20)
*mhcI*	GACAGTCCGTCCCTCAGTGT	CTGGAAGGTTCCATCATCGT		(21)
*mhcII*	TGCCATGCTGATGTGCAG	GTCCCTCAGCCAGGTCACT	CGCCTATGACTTCTACCCCAAACAAAT	(22)

### Immunofluorescence Assays

Purified RBCs were fixed as previously described (14), using 4% paraformaldehyde (PFA; Sigma-Aldrich) and 0.008% glutaraldehyde (GA, Sigma-Aldrich) in RPMI medium. Anti-MX (25, 26) and anti-IL8 (27) were used as primary antibodies and goat-CF™647 anti-mouse IgG (H+L) and goat-CF™647 anti-rabbit IgG (H+L) antibodies (Sigma-Aldrich) were used as secondary antibodies. Nuclear staining was performed with 1 μg/mL of 4′-6-Diamidino-2-phenylindole (DAPI, Sigma-Aldrich). Images were captured in an IN Cell Analyzer 6000 Cell Imaging system. Flow cytometry was carried out in a FACSCanto™ flow cytometer.

### Software and Statistics

Graphpad Prism 6.01 (www.graphpad.com) was used for statistics and graphic representation. Statistic tests and *P*-values associated with graphics are indicated in each assay. Flow cytometry data was processed and analyzed using Flowing Software 2.5.1 (www.flowingsoftware.com/). Principal component analysis (PCA) and clustering of gene expression analysis was performed using ClustVis software (https://biit.cs.ut.ee/clustvis/) (24).

## Results

### Uptake of IB^TNFα^ and IB^frg16G-VHSV^ by RBCs

**Figure 1:**
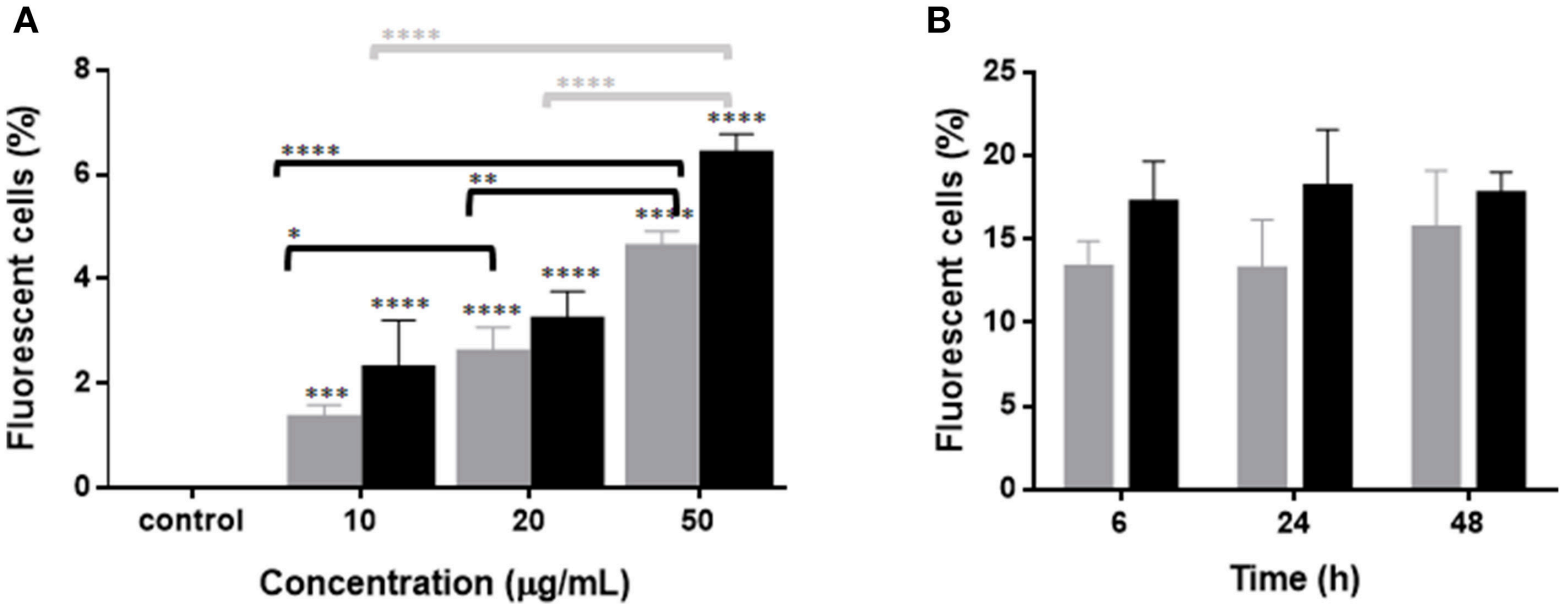
Uptake of IB^TNFα^ and IB^frg16G−VHSV^ by RBCs *in vitro*. **(A)** Dose-response of RBCs incubated 24 h with 10–50 μg/mL IB^frg16G−VHSV^ (gray bars) or IB^TNFα^ (black bars). **(B)** Time course monitoring of RBCs incubated 6, 24, and 48 h with 160 μg/mL IB^frg16G−VHSV^ (gray bars) or 80 μg/mL IB^TNFα^ (black bars). Data represent mean ± SD (*n* = 4). Two-way Anova and Dunnett's multiple comparisons test was performed between all conditions and control (untreated cells) and among concentrations. ^*^, ^**^, ^***^, ^****^P-value < 0.05, 0.01, 0.001, and 0.0001, respectively.

**Figure 2:**
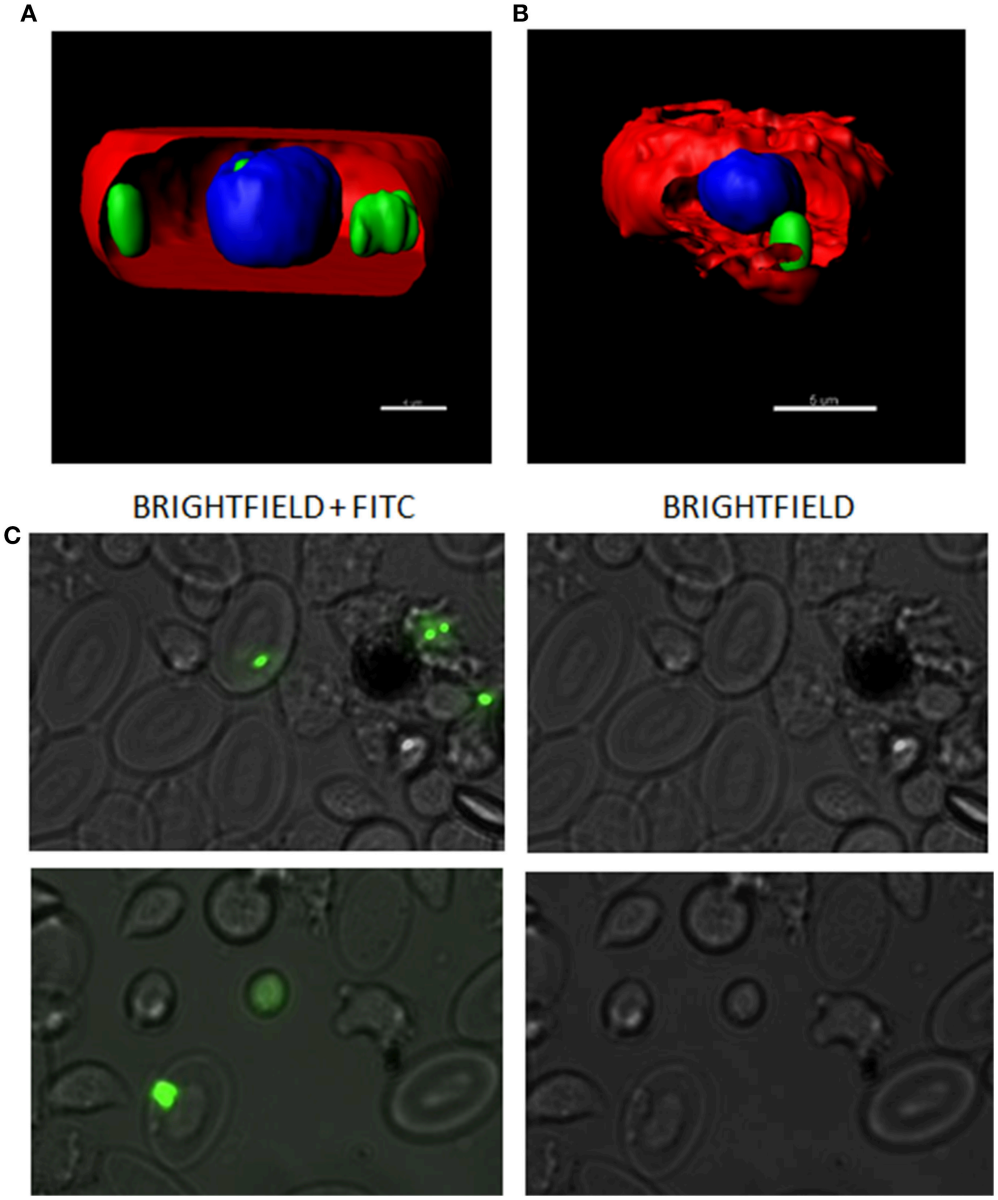
Confocal microscopy images digitalized using z-stack. RBCs incubated with **(A)** 80 μg/mL IB^TNFα^ or **(B)** 160 μg/mL IB^frg16G−VHSV^ for 24 h. IBs are showed in green, cell membrane (CellMask™) in red and nucleus (Hoechst-stained) in blue. **(C)** IBs monitorization *in vivo* in cells obtained from head kidney of rainbow trout injected intravenously with 5.5 mg/kg of IB^TNFα^, 3 h post- injection. Representative bright-field and FITC microscopy images taken with 40× magnification.

In order to evaluate the interaction between RBCs and IBs, we performed a dose-response and time-course evaluation by means of flow cytometry. According to our results, all IB concentrations assayed showed uptake or attachment to RBCs, which increased with IB concentration (Figure 1A). The percentage of IB positive cells ranged from 5 to 7% at 50 μg/mL after 24 h incubation. Time course evaluation at 6, 24, and 48 h showed no differences in IB load in RBCs (Figure 1B) indicating that the maximum IB internalization or attachment occurred by 6 h of incubation. However, the time course was carried out with a higher dose and up to 17% of fluorescent positive cells were detected. This was maximum percentage uptake achieved under our experimental conditions. The level of uptake of IB^TNFα^ by RBCs was observed to be higher than IB^frg16G−VHSV^ when comparing the same concentration of both IBs (Figure 1A). IB uptake was confirmed by confocal 3D images, which showed the internalization of IB^TNFα^ (Figure 2A) and IB^frg16G−VHSV^ (Figure 2B) in the cytosol of RBCs.

The presence of IB^TNFα^ in RBCs was monitored *in vivo* in peripheral blood and head kidney cells by fluorescent microscopy using intravenously injected IB^TNFα^. In blood, few RBCs were found to carry the IB^TNFα^ (data not shown); however, RBCs carrying IB^TNFα^ were easily found in head kidney cells extracts (Figure 2C).

### Immune Response of RBCs Induced After Exposure to IB^TNFα^ or IB^frg16G-VHSV^ *in vitro*

To explore the immune response triggered by IBs in RBCs *in vitro*, RBCs were treated with 50 μg/mL of IB^TNFα^, IB^frg16G−VHSV^ or IB^iRFP^ and RNA was extracted at 24 h post-treatment. IB^TNFα^ tended to down-regulate the genes tested in RBCs at 24 h post-treatment. This down-regulation was statistically significant in genes related to antigen presentation (*cd83, mhcI*) and antioxidant gene *gstp1*. On the other hand, only the antioxidant *trx* gene was significantly up-regulated in IB^frg16G−VHSV^ treated RBCs at 24 h post-treatment (Table 2).

**Table 2:** Immune-gene expression analysis of RBCs stimulated *in vitro* with 50 μg/mL of IB^iRFP^, IB^TNFα^ and IB^frg16G−VHSV^ at 24 h post-treatment.

	**IB^TNFα^**		**IB^frg16G−VHSV^**	
	**Mean**	**SD**	**Mean**	**SD**
*mx*	0.902	0.157	1.013	0.199
*il15*	0.943	0.288	1.181	0.414
*cd83*	0.782^***^	0.042	0.918	0.101
*mhcI*	0.794^*^	0.138	0.899	0.145
*mhcII*	0.965	0.235	1,270	0.428
*nkef*	1.106	0.753	1.067	0.943
*gstp1*	0.785^**^	0.105	1.254	0.588
*trx*	1.070	0.179	1.289^**^	0.316
*tlr3*	0.866	0.163	0.887	0.198
*tlr9*	0.814	0.656	0.907	0.623

*RBCs were Ficoll-purified and treated with IBs. 24 h post-treatment gene expression was analyzed by RT-qPCR, 2^−ΔΔCt^ method, normalized to the endogenous gene eukaryotic 18S, and relative to control cells (treated with IB^iRFP^). Data represent mean fold change± SD (n = 4). Mann-Whitney test was performed between each condition and control cells*.

*, **, *** *P-value < 0.05, 0.01, and 0.001 respectively*.

**Figure 3:**
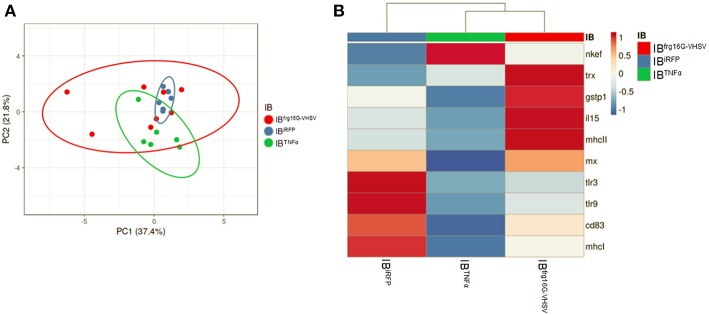
Principal component analysis (PCA) of immune-gene expression evaluation of RBCs stimulated *in vitro* with 50 μg/mL of IB^iRFP^, IB^TNFα^, or IB^frg16G−VHSV^, at 24 h post-treatment. **(A)** PCA plot of molecular (gene expression data, 2^−ΔΔCt^) signatures of IB^iRFP^, IB^TNFα^, or IB^frg16G−VHSV^ treated samples, at 24 h post-treatment. Ellipses and shapes show clustering of the samples. **(B)** Heatmap of molecular (gene expression data, 2^−ΔΔCt^) signatures of IB^iRFP^, IB^TNFα^, or IB^frg16G−VHSV^ treated samples. Annotations on top of the heatmap show clustering of the samples, mean values. PCA plot and heatmap were performed using Clustvis software. Heatmap data matrix visualizes the values in the cells using a color gradient which gives an overview of the largest and smallest values in the matrix.

In order to analyse the gene expression of RBCs in response to each treatment as a whole, multivariate analyses of the gene expression data matrix were performed. A principal component analysis (PCA) plot of the gene expression profile showed a differentiated population of RBCs treated with IB^TNFα^ or IB^frg16G−VHSV^ compared to IB^iRFP^ (Figure 3A). This is also appreciable in the clustering heatmap (Figure 3B), where the mean values of molecular (gene expression) signatures are clustered. The heatmap data matrix visualizes the values in the cells by the use of a color gradient which gives an overview of the largest and smallest values in the matrix (24).

### Immune-Gene and Protein Expression Modulation in RBCs From Peripheral Blood After *in vivo* Treatment With IB^TNFα^ or IB^frg16G-VHSV^

**Figure 4:**
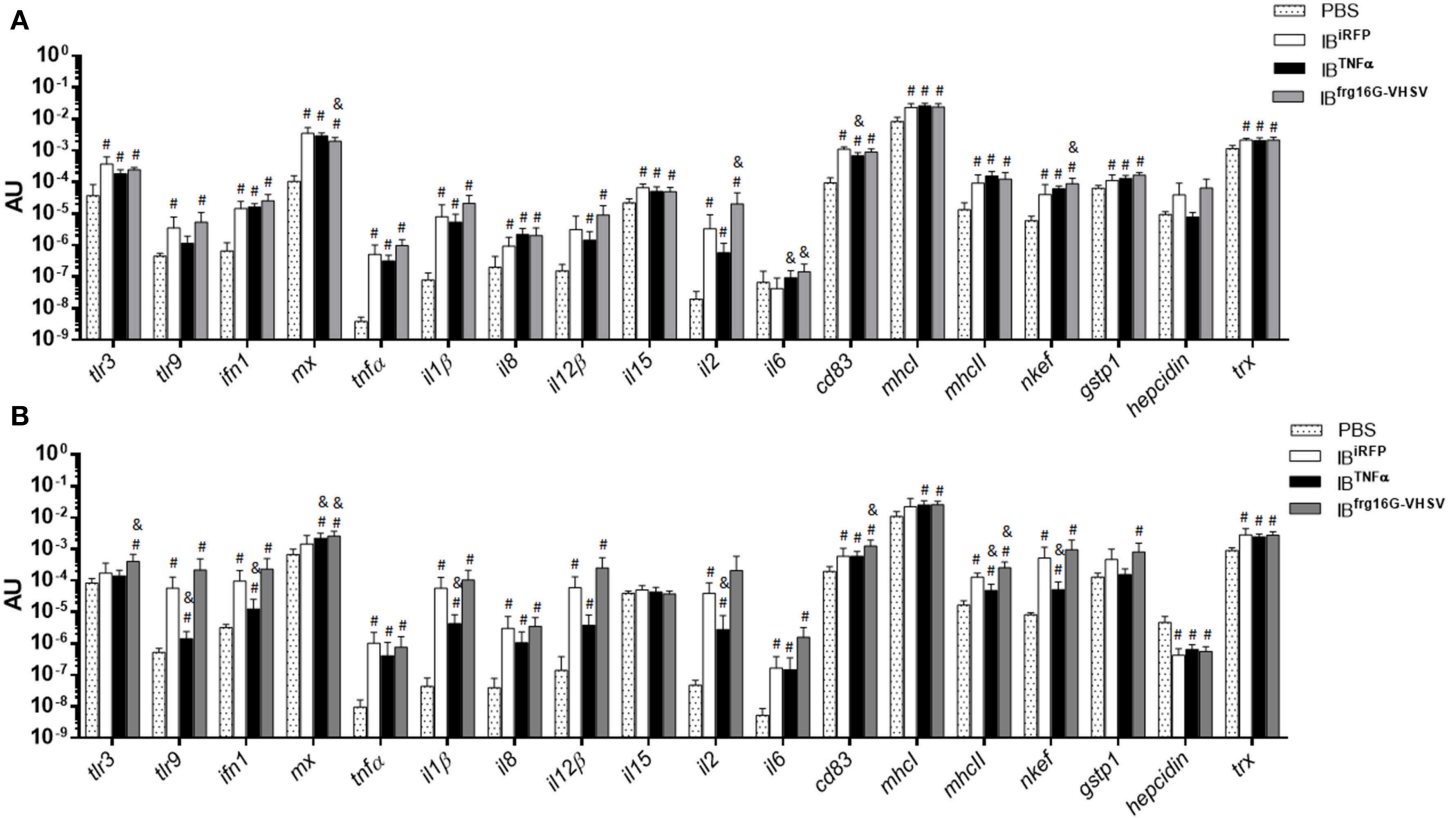
Immune-gene expression analysis of RBCs from rainbow trout injected intravenously with PBS, IB^iRFP^, IB^TNFα^, or IB^frg16G−VHSV^ at 24 and 48 h post-injection. Rainbow trout of 15–20 g were injected with 5.5 mg/kg of IB intravenously. Blood was extracted and RBCs Ficoll-purified 24 h **(A)** and 48 h **(B)** post-injection. Gene expression was analyzed by RT-qPCR, 2^−ΔCt^ method, with endogenous gene eukaryotic 18S rRNA. Data represent mean AU (arbitrary units) ± SD (*n* = 4). Mann-Whitney test was performed between each condition and control (treated with PBS or IB^iRFP^). #*P*-value < 0.05, compared to PBS; & *P*-value < 0.05, compared to IB^iRFP^.

Rainbow trout were intravenously injected to evaluate the immune response triggered by IBs in RBCs of peripheral blood *in vivo*. RBCs were sampled at 24 and 48 h post-injection. In general, the results showed, as *in vitro*, a down-regulatory trend in the gene expression of IB^TNFα^ treated individuals compared to IB^iRFP^ treated individuals. It should be noted that *cd83* was significantly down-regulated at 24 h post-injection (Figure 4A), as occurred *in vitro*. On the other hand, *il6* was significantly up-regulated at 24 h post-injection. Further, *tlr9, ifn1, il1*β*, il2, mhcII* and *nkef* genes were significantly down-regulated at 48 h post-injection (Figure 4B). In contrast, IB^frg16G−VHSV^ treated individuals showed an up-regulatory trend at both 24 and 48 h post-injection, compared to IB^iRFP^, with significant up-regulation of cytokines *il2* and *il6*, and antioxidant gene *nkef* at 24 h post-injection, and of *tlr3*, interferon inducible *mx, cd83*, and *mhcII* at 48 h post-injection (Figures 4A,B, Table S1). However, *mx* gene appeared down-regulated at 24 h post-injection. Separately, most of the genes were up-regulated with all the treatments in comparison with PBS-injection.

**Figure 5:**
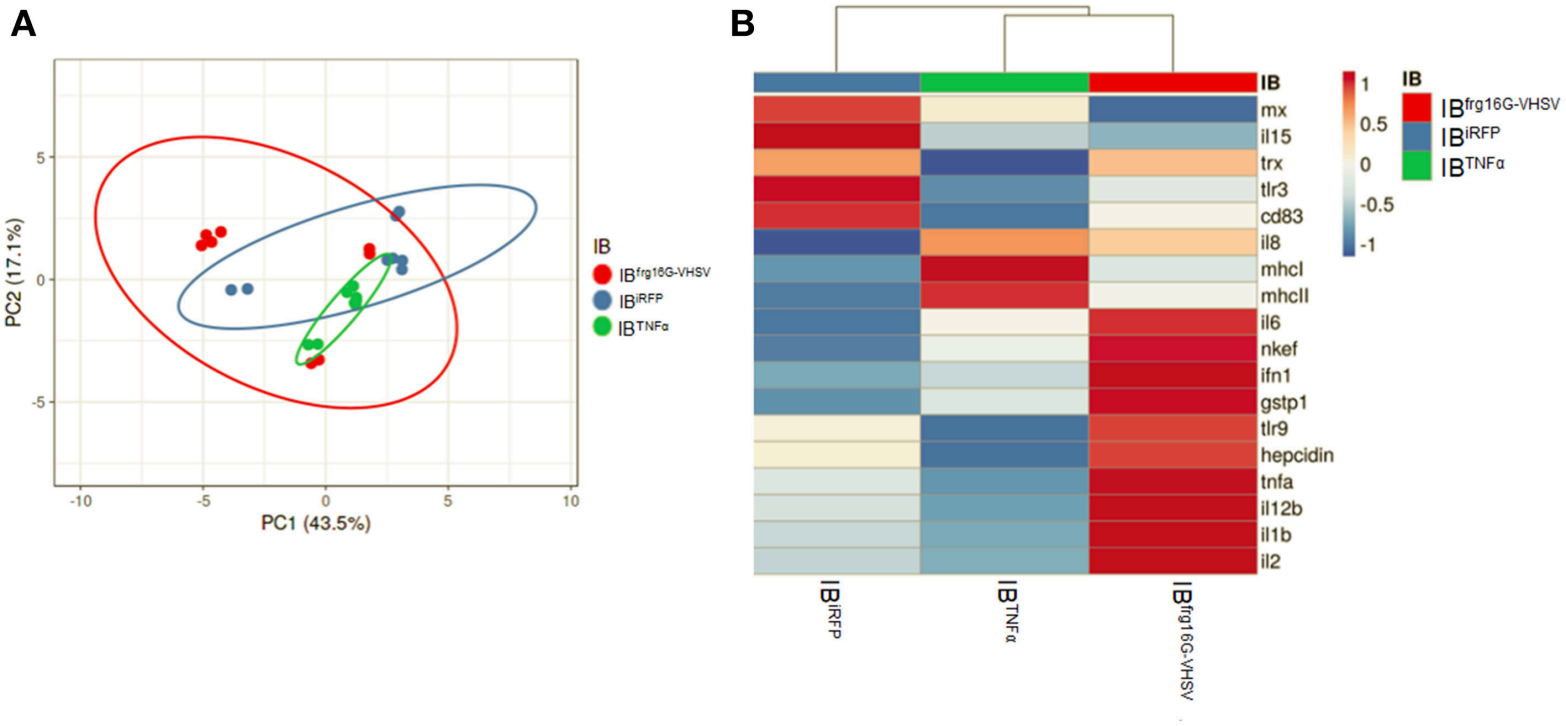
Principal component analysis (PCA) of immune-gene expression evaluation of RBCs from rainbow trout injected intravenously with IB^iRFP^, IB^TNFα^, or IB^frg16G−VHSV^, at 24 h post-injection. **(A)** PCA plot of molecular (gene expression data, 2 ^−ΔCt^) signatures footprint of IB^iRFP^, IB^TNFα^, or IB^frg16G−VHSV^ treated samples, at 24 h post-injection. Ellipses and shapes show clustering of the samples. **(B)** Heatmap of gene expression (2 ^−ΔCt^) signatures of IB^iRFP^, IB^TNFα^, or IB^frg16G−VHSV^ treated samples. Annotations on top of the heatmap show clustering of the samples mean values. PCA plot and heatmap was performed using Clustvis software. Heatmap data matrix visualizes the values in the cells using a color gradient which gives an overview of the largest and smallest values in the matrix.

**Figure 6:**
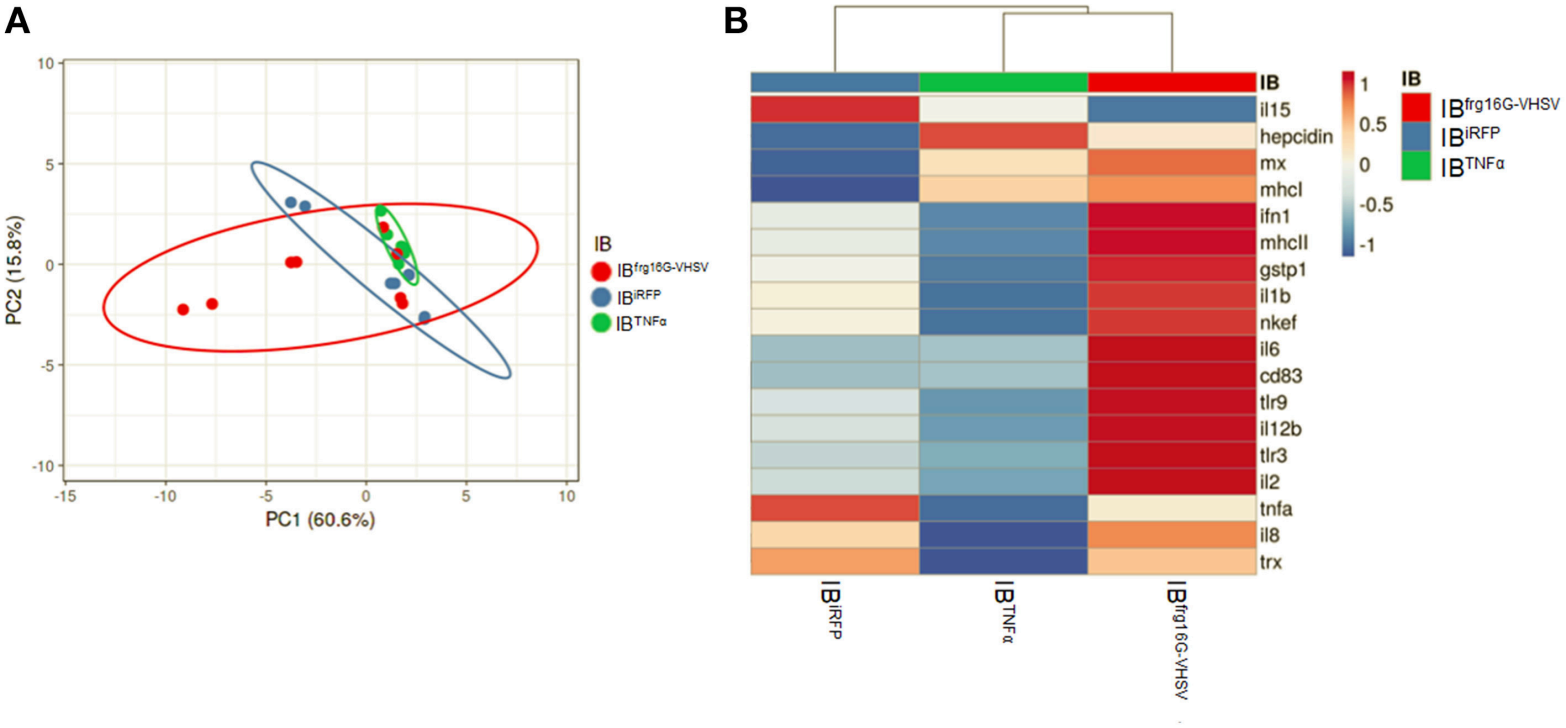
Principal component analysis (PCA) of immune-gene expression evaluation of RBCs from rainbow trout injected intravenously with IB^iRFP^, IB^TNFα^, or IB^frg16G−VHSV^, at 48 h post-injection. **(A)** PCA plot of molecular (gene expression data, 2 ^−ΔCt^) signatures of IB^iRFP^, IB^TNFα^, or IB^frg16G−VHSV^ treated samples, at 48 h post-injection. Ellipses and shapes show clustering of the samples. **(B)** Heatmap of molecular (gene expression data, 2 ^−ΔCt^) signatures of IB^iRFP^, IB^TNFα^, or IB^frg16G−VHSV^ treated samples. Annotations on top of the heatmap show clustering of the samples mean values. PCA plot and heatmap was performed using Clustvis software. Heatmap data matrix visualizes the values in the cells using a color gradient which gives an overview of the largest and smallest values in the matrix.

**Figure 7:**
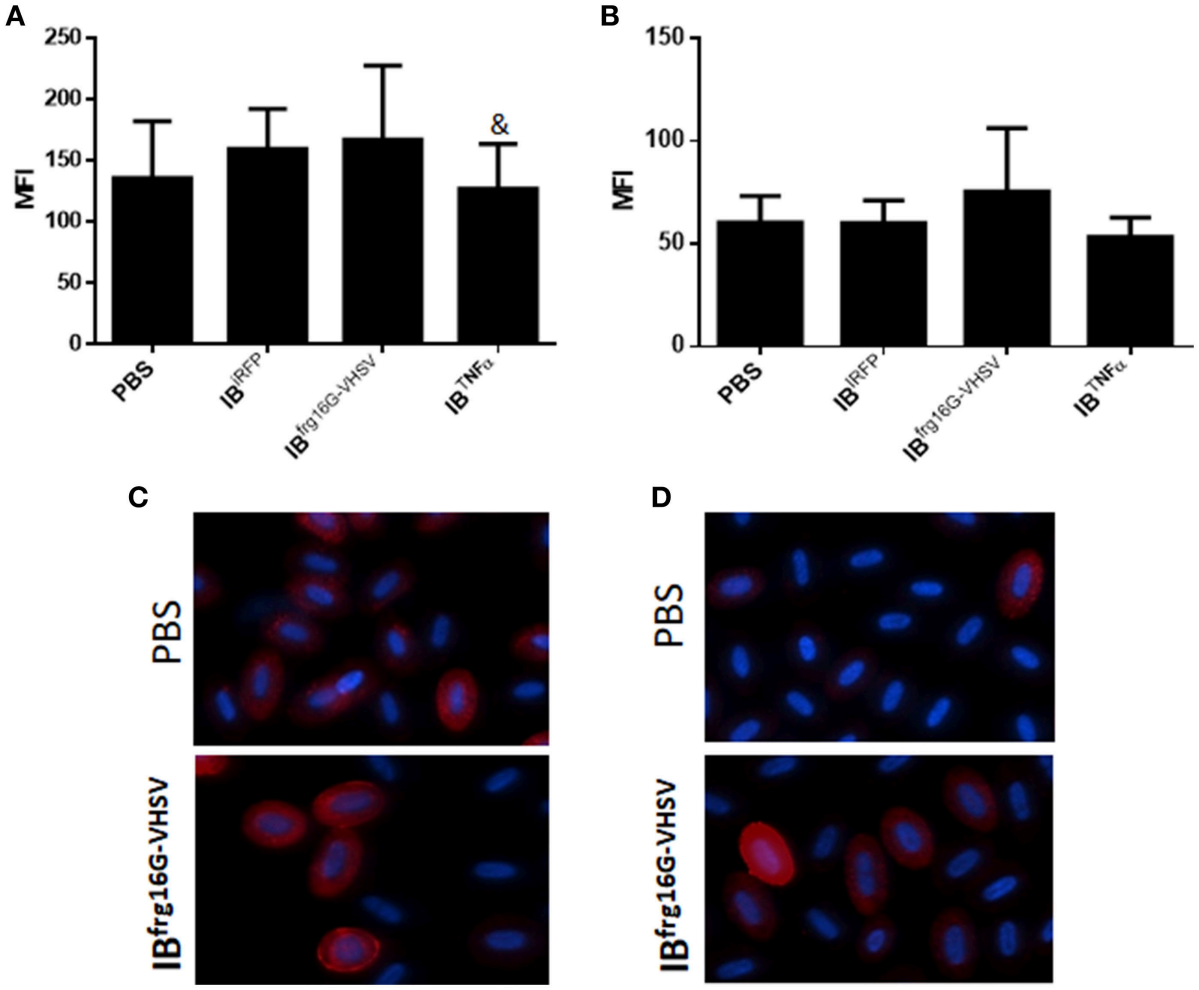
Protein expression analysis of RBCs from rainbow trout injected intravenously with 5.5 mg/kg of IB^iRFP^, IB^TNFα^, IB^frg16G−VHSV^ or PBS at 48 h post-injection. **(A)** Interferon related protein MX and **(B)** chemokine IL8 Mean Fluorescence Intensity (MFI) measured by flow cytometry. Data represent mean ± SD (*n* = 4). Mann-Whitney test was performed between each condition and control cells (treated with PBS or IB^iRFP^). &*P*-value < 0.05, compared to IB^iRFP^. Representative immunofluorescence images of RBCs stained with **(C)** anti-MX and **(D)** anti-IL8, taken with 60× magnification. Protein stain in red, DAPI (blue) for nuclei stain.

**Figure 8:**
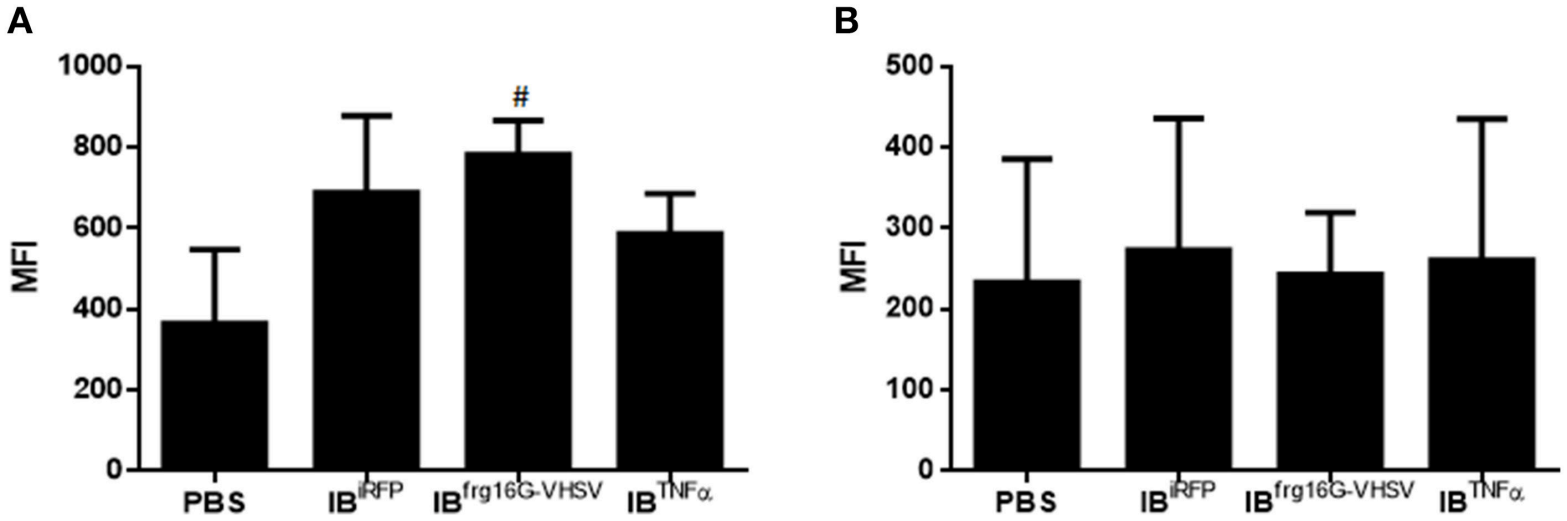
Protein expression analysis of total blood samples from rainbow trout injected intravenously with 5.5 mg/kg of IB^iRFP^, IB^TNFα^, IB^frg16G−VHSV^, and PBS at 48 h post-injection. **(A)** Interferon related protein MX and **(B)** chemokine IL8 Mean Fluorescence Intensity (MFI) measured by flow cytometry. Data represent mean ± SD (*n* = 4). Mann-Whitney test was performed between each condition and control cells (treated with PBS or IB^iRFP^). #*P*-value < 0.05, compared to PBS.

The gene expression profile PCA plot depicted differentiated populations for RBCs from individuals treated with IB^TNFα^ or IB^frg16G−VHSV^ compared to IB^iRFP^ (Figures 5A, 6A, for 24 and 48 h post-injection, respectively), which was also observed in the clustering heatmap (Figures 5B, 6B, for 24 and 48 h post-injection respectively). In addition, at 48 h post-injection, MX and IL8 protein levels, evaluated by means of flow cytometry, showed an increment, but not statistically significant, in MX (Figures 7A,C) and IL8 (Figures 7B,D) in RBCs from rainbow trout treated with IB^frg16G−VHSV^ in relation to PBS-injected or the other IBs assayed. This result correlates with the *mx* gene expression at 48 h *in vivo*. On the other hand, the protein levels of MX and IL8 in RBCs from IB^TNFα^ treated rainbow trout were slightly lower than IB^iRFP^ and PBS-injected individuals (only showing statistical significance for MX between IB^TNFα^ and IB^iRFP^ treatments), which is consistent with the down-regulatory trend observed in IB^TNFα^ treated RBCs *in vivo* and *in vitro* at the transcriptional level. Moreover, in whole peripheral blood, a similar tendency was observed in MX protein expression, although more pronounced in this case. Note, however, for IL8 protein levels, we did not observe any difference among groups (Figures 8A,B).

## Discussion

Recently, IBs have been reported as new alternatives in fish prophylaxis as immunostimulants or adjuvants (10), thus potentially serving as a new platform for vaccine delivery. The uptake of IBs has been reported in rainbow trout macrophages (RT-HKM) and zebrafish liver cells (ZFL). In both cell types IBs made with cytokines stimulate the innate immune response (9). Moreover, IBs made with fish viral antigens have evoked an anti-viral innate immune response in ZFL and RT-HKM (12). However, the immune response of nucleated RBCs to nanostructured cytokine or viral antigen IBs has not been tested until now. Nucleated RBCs are the main cell in the blood and recently have been endorsed as immune cells mediators (6, 28). In this work we show that the uptake or attachment of IBs by rainbow trout RBCs occurred in approximately 7% of cells counted. This contrasts to the near 40% and 80% reported for ZFL and RT-HKM, respectively, at same concentration (50 μg/mL) of IB^TNFα^ (9). RBCs endocytosed both the IBs tested here reaching their maximum level at 6 h post-treatment, in contrast to RT-HKM and ZFL cell lines, which reached their maximum uptake at 24 h post-treatment *in vitro* (10). Besides, monitorization of IB^TNFα^ *in vivo* demonstrated its presence on/in RBCs from head-kidney 3 h post-injection. The mechanism by which RBCs endocytose IBs is unknown. It may occur via the micropinocytosis endocytic pathway, as proposed for mammalian cells (29).

Significantly, with this work, we add to the growing body of data demonstrating nucleated RBCs can exercise a role in the immune response. RBCs are able to respond to virus (6, 14, 30), produce cytokines when exposed to stimuli (17), and endocytose pathogens (2). Here we show for the first time rainbow trout RBCs evoke an immune response to IBs made of cytokine TNFα and viral protein frg16G-VHSV *in vitro* and *in vivo*. We demonstrate this response at protein and transcript level. Rainbow trout Ficoll-purified RBCs treated with IBs *in vitro* and RBCs Ficoll-purified from blood extracts from IB-intravenously injected individuals modulated the expression of genes related to antigen presentation, cytokines and other genes involved in the immune response. PCA clearly clustered the RBCs' immune-gene expression profiles for each treatment.

As regards TNFα, RBCs from IB^TNFα^-treated rainbow trout individuals showed a down-regulatory trend for genes related to TNFα signaling such as *tlr9, tnf*α, *il1*β, *il12*β, and *il2* genes transcripts, *in vivo*, at 24 and 48 h post-injection, compared to fish injected with the non-immunogenic protein IB^iRFP^. It is known that TNFα is a cytokine involved in the regulation of immune cells and inflammation. It is mainly produced by monocytes and macrophages along with additional producers including B and T lymphocytes, NK cells, polymorphonuclear leukocytes, and eosinophils in response to bacterial toxins, inflammatory products, and other invasive stimuli (31). Recently, nucleated RBCs have been also reported to modulate TNFα protein in response to IPNV virus exposure (30). Here we observed that RBCs exposed to IB^TNFα^ down-regulated the inflammatory response at 24 and 48 h post-treatment. TNFα is a pleiotropic cytokine with a diverse range of biological actions. TNF family members are known to represent a “double-edged sword,” having both beneficial and detrimental activities (32). Systemic exposure to recombinant TNFα would cause a shock similar to septic shock syndrome (31). Further, TNFα inhibition of IFNγ-induced IL12 production exerts mechanisms by which TNFα and IL12 cytokines can elicit anti-inflammatory and repair functions, tightly modulated by positive and negative feedback signals for optimal immunity without manifested inflammation (33). Another important observation is that fish recombinant TNFα has been reported to regulate the expression of endothelial cells TLRs, including TLR9, but had negligible effects on macrophages (34). Therefore, taking into account that nucleated RBCs are the most abundant cell type in peripheral blood, it would make sense that RBCs were equipped to modulate inflammation in response to a systemic exposure to TNFα. Moreover, in the IB^TNFα^ injected group, genes related to antigen presentation, *cd83* and *mhcII*, were also down-regulated at 24 and 48 h, respectively. As well, RBCs treated *in vitro* with IB^TNFα^ down-regulated the expression of *cd83* and *mhcI* 24 h post-treatment. TNFα has been reported to modulate IFNγ-induced MHC class II expression in a cell type-specific mode (35). Therefore, TNFα treatment augments or blocks MHC class II induction depending on the cell type and cellular differentiation state (35). *mhcII* and *cd83* gene expression has been previously reported for rainbow trout RBCs (3, 36) and chicken RBCs (37). However, this is the first report that shows the regulation of *cd83* and *mhcII* gene transcripts in response to an immunostimulant.

On the other hand, RBCs from rainbow trout injected with IB^frg16G−VHSV^ showed an up-regulatory trend for most of the genes, specifically interleukins *il2* and *il6*, and antioxidant enzyme *nkef* were significantly up-regulated, compared to IB^iRFP^, at 24 h post-injection. This is probably due to the effort of RBCs to compensate the inflammatory response triggered after the first treatment stimulus. Then, 48 h post-injection, the Type 1 IFN and antigen presentation responses were increased, since *tlr3, mx, cd83*, and *mhcII* genes transcripts were significantly up-regulated, compared to IB^iRFP^. MX protein production was consistent with gene expression levels.

G-VHSV is known to induce the expression of *ifn1* and *mx* (25, 38, 39). Peptides derived from G-VHSV have also demonstrated their efficacy to induce type 1 IFN response (25, 26, 39). It is also noteworthy that IB^frg16G−VHSV^ triggered the up-regulation of *mhcII* and *cd83* gene expression in rainbow trout RBCs, thus endowing them the characteristics of antigen presenting cells (APCs). CD83 and MHCII are principally produced by professional APCs to process antigens and induce T cell priming. However, recently, the concept of non-professional APCs is emerging (40). These atypical APCs up-regulate the expression of MHC and related molecules under certain stimuli. However, there is not enough evidence about their functionality priming T cells (40).

Bacterial lipopolysaccharide has been reported to stimulate the innate immune response of RBCs *in vitro* (28). Bacterial IBs, which contain remnants of endotoxin, are therefore considered immunostimulants *per se* (41), which is shown by the global increment in the immune response of RBCs from rainbow trout injected with IB^iRFP^ compared to PBS-injection. This, added to the utilization of IBs as delivery platforms to administrate cytokines, coadjuvants, or antigens, makes them a good candidate for future vaccines. In this context, RBCs have shown their ability to mount or modulate and immune-response to IBs made of cytokine TNFα and the viral protein frg16G-VHSV.

All these considerations provide a new perspective on the role and potential use of RBCs. Given the large amount of RBCs in the organism and their rapid distribution throughout the body they could be a promising target cell for the presentation or delivery of IBs or other types of vaccine carriers.

## Ethics Statement

Experimental protocols and methods of the experimental animals at the UMH were reviewed and approved by the Animal Welfare Body and the Research Ethics Committee at the University Miguel Hernandez (approval number 2014.205.E.OEP; 2016.221.E.OEP) and by the competent authority of the Regional Ministry of Presidency and Agriculture, Fisheries, Food and Water supply (approval number 2014/VSC/PEA/00205). All methods were carried out in accordance with the Spanish Royal Decree RD 53/2013 and EU Directive 2010/63/EU for the protection of animals used for research experimentation and other scientific purposes. All experimental procedures of the experimental animals at the UAB were approved by the Human and Animal Experimentation Ethics Committee of the Universitat Autònoma de Barcelona (Reference 1533) and were done in strict accordance with the recommendations of the European Directive (2010/63/EU) on the protection of animals used for scientific purposes.

## Author Contributions

SP-M performed experiments, analyzed data, and wrote the manuscript. RT performed experiments and contributed to manuscript preparation. JC contributed to IB^frg16G−VHSV^ construction. LM provided valuable antibodies for the experiments. NR conceived ideas, oversaw the research and contributed to manuscript preparation. MO-V conceived ideas, oversaw the research and co-wrote the manuscript.

### Conflict of Interest Statement

The authors declare that the research was conducted in the absence of any commercial or financial relationships that could be construed as a potential conflict of interest.
