# Supplementary figures and images for: Fish Red Blood Cells Modulate Immune Genes in Response to Bacterial Inclusion Bodies Made of TNFα and a G-VHSV Fragment
Source: Front Immunol. 2019 May 22;10:1055. doi: 10.3389/fimmu.2019.01055 (PMC6538768; doi:10.3389/fimmu.2019.01055)

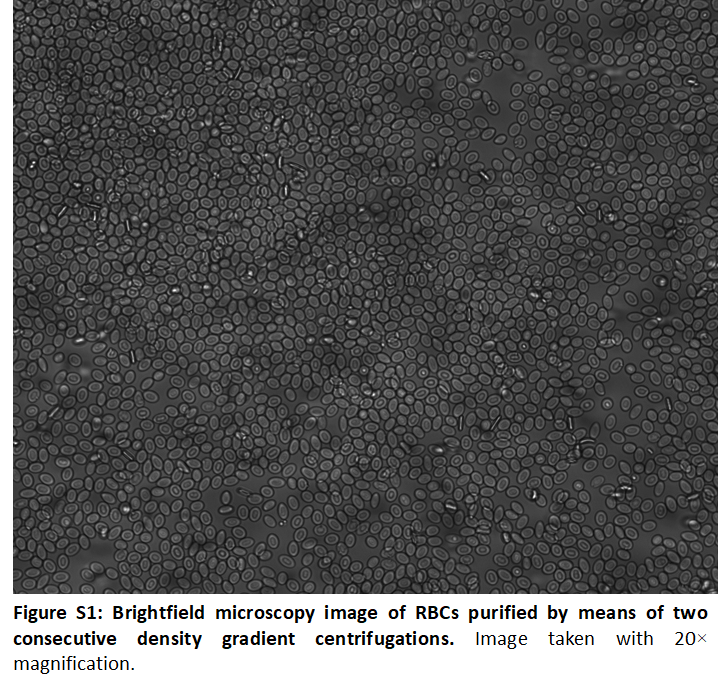

Supplement: Supplementary file 2 [file Image_1.TIF]
